# Insights into protection against *Mycobacterium tuberculosis* infection: time to officially confirm another phenotype?

**DOI:** 10.1172/JCI191423

**Published:** 2025-04-01

**Authors:** Todia P. Setiabudiawan, Philip C. Hill, Andrew R. DiNardo, Reinout van Crevel

**Affiliations:** 1 Department of Internal Medicine and Radboud Community for Infectious Diseases, Radboud University Medical Center, Nijmegen, Netherlands.; 2 Research Center for Care and Control of Infectious Diseases, Universitas Padjadjaran, Jawa Barat, Indonesia.; 3 Centre for International Health, University of Otago, Dunedin, New Zealand.; 4 The Global Tuberculosis Program, Texas Children’s Hospital, Department of Pediatrics, Baylor College of Medicine, Houston, Texas, USA.; 5 Centre for Tropical Medicine and Global Health, Nuffield Department of Medicine, University of Oxford, Oxford, United Kingdom.

## Abstract

Immune correlates of protection against infection with *Mycobacterium* *tuberculosis* (Mtb) remain elusive. In this issue of the *JCI*, Dallmann-Sauer and authors demonstrate that lack of tuberculin skin test (TST) and interferon γ release assay (IGRA) conversion among people with HIV despite years-long Mtb exposure is associated with alveolar lymphocytosis, including specific poly-cytotoxic T cells, and M1-type alveolar macrophages with a stronger ex vivo response to the pathogen. Studies in these rare individuals, termed “TB resisters” and in tuberculosis household contacts who are repeatedly IGRA negative in the months after a specific exposure event (known as “early clearers”) help elucidate manipulatable mechanisms to boost protection against Mtb infection.

## *Mycobacterium tuberculosis* exposure versus infection and TB disease

**Figure 1:**
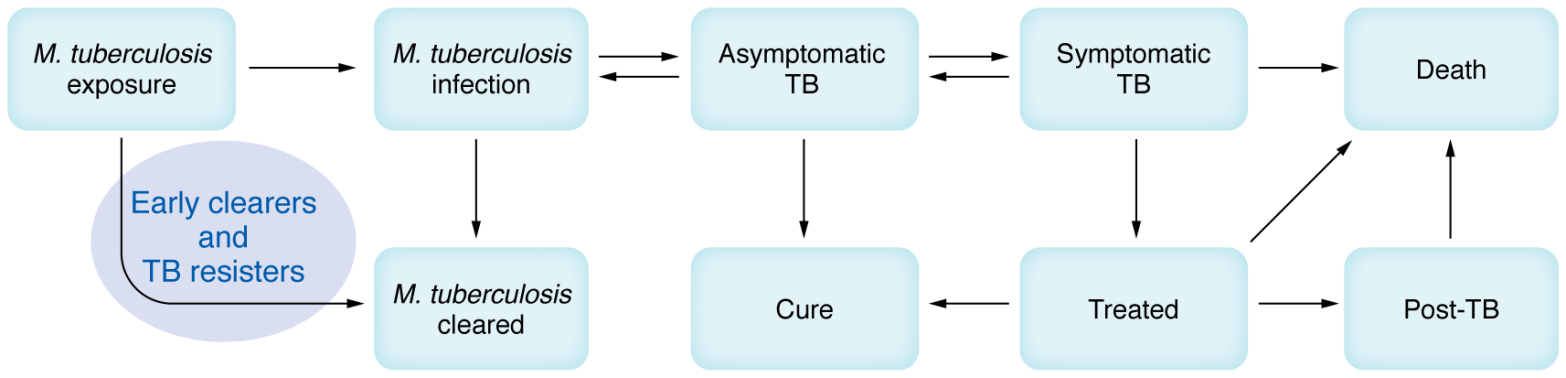
Different stages after exposure to Mtb. The TB resister phenotype is characterized by persistently negative IGRA/TST despite years-long exposure whereas early clearance reflects repeatedly negative IGRA results over a short period (e.g., 3 months) in the context of a well-defined exposure of TB household contacts to an index patient with a known Mtb isolate.

In recent years, the historic paradigm of active disease versus latent tuberculosis (TB) infection has been replaced by a more nuanced view on the natural course of events after exposure to *Mycobacterium* *tuberculosis* (Mtb), blurring the boundaries and movement between phenotypes in the individual. A range of terms has been used to describe early TB disease states and frameworks have been developed (1). Early, asymptomatic, stages of TB disease appear to be important for individual patient health and Mtb transmission. WHO has formally distinguished asymptomatic from symptomatic TB accordingly and bacteriologically confirmed from unconfirmed disease (2). The period termed “post-TB” is also increasingly recognized, as patients who are microbiologically cured are at increased risk of persistent lung disease, secondary infections, cardiovascular events, and malignancies (3), causing as much as 50% of TB-associated morbidity and mortality (3). An emerging, but perhaps not officially confirmed, phenotype is one where Mtb is cleared rather than being able to establish a lasting infection (Figure 1). Supporting this concept, strong experimental evidence from a nonhuman primate Mtb infection model demonstrates that complete bacterial clearance can occur (4, 5).

The tuberculin skin test (TST) and interferon γ release assay (IGRA), which are used to indicate Mtb infection, are indirect and cannot distinguish mere T cell immunoreactivity and true Mtb infection. As much as a quarter of the world’s population may be immunoreactive, but recent modeling estimates that only 2% (about 160 million people) carry viable Mtb, with others likely to have cleared the pathogen over time (6). Interestingly, some people, termed “TB resisters,” do not develop a positive TST or IGRA (nor TB disease), even when exposed to patients with TB repeatedly over many years (7). Obviously, better insight into the mechanisms underlying this protection could help develop or improve TB preventive strategies.

## What happens in the lung of TB resisters

So far, studies searching for correlates of protection against Mtb infection have looked at blood and not at the lung as the site of infection. They have shown altered innate immune responses (8, 9), IFN-γ independent T cell responses (10–12), Mtb-specific antibody production (13, 14), and genetic traits (15). In this issue of the *JCI*, Erwin Schurr’s group explored local immune correlates in the lung (16). They used single-cell RNA-Seq on bronchoalveolar lavage from seven TB resisters with a stable and well-controlled HIV infection and a control group of people with HIV and sustained TST and IGRA positivity. Compared with TST/IGRA-positive individuals, TB resisters had a higher abundance of alveolar lymphocytes, with a remarkable increase of CD8^+^ polycytotoxic T cells that coexpress the key antimicrobial molecules granulysin, granzyme B, and perforin. A specific subpopulation of these polycytotoxic T cells also showed increased expression of genes for NK cell receptors, particularly NKG2D. Alveolar macrophages had a more M1-like transcriptional signature, characterized by higher expression of proinflammatory genes IL6, CCL3, and IL1B, while showing reduced expression of the M2 marker CD163. Upon ex vivo Mtb challenge, macrophages from TB resisters demonstrated stronger TNF signaling and notably higher upregulation of MHC class I polypeptide–related sequence A (MICA) transcripts, which serve as ligands for the NK cell receptors. The alveolar lymphocytes from TB resisters demonstrated increased basal, unstimulated IFNG levels, an interesting phenotype since they seem to demonstrate a constitutive activated state, without leading to exhaustion. Constitutive immune overactivation results in immune exhaustion (17) and increased infection risk, yet these data suggest that resister lymphocytes were able to remain in an immune-activated state without becoming exhausted.

Based on a large amount of collected data, Dallmann-Sauer and authors speculated about the interplay of T cells and alveolar macrophages in TB resisters, proposing that constitutive IFNG expression from T cells combined with alveolar lymphocytosis leads to increased IFN-γ levels, which drive alveolar macrophages toward an M1-like state, priming them for enhanced antimicrobial responses (14, 16). Upon Mtb infection, these primed macrophages mount a stronger TNF response, triggering cellular stress and upregulation of MICA, a ligand for the NK-cell receptor NKG2D. MICA is highly expressed on polycytotoxic T cells, allowing them to recognize and eliminate Mtb-infected macrophages more efficiently. Clearly, this hypothetic model needs validation in other cohorts and in experimental animal models that reflect the TB-resister state. Additionally, this is unlikely to be the only molecular pathway leading to resistance against Mtb infection.

Dallmann-Sauer’s study (16) was conducted in individuals living with HIV, which is an interesting choice. Individuals with HIV who live in a TB-endemic area but do not develop TB provide a model of natural resistance against TB, and a study from Uganda and Tanzania has identified genetic loci associated with this protection (18). On the other hand, lack of T cell immunoreactivity (a negative TST or IGRA) in people living with HIV may also reflect T cell anergy after Mtb exposure, which might lead to misclassification of the TB resister phenotype. Of note, TB resisters in this study had detectable anti-Mtb antibodies. It remains to be determined if the single-cell data from alveolar lavage in these study subjects can be extrapolated to HIV-uninfected individuals.

## TB resisters and early clearers

The TB resister phenotype, characterized by a negative TST and IGRA over a long period of exposure (13), has similarities to what our group has termed “early clearance” (19). The word clearance draws from the historical concept of “delayed clearance,” which was used to describe the small but steady cumulative increase in TST reversions in initially TST-positive individuals over decades after Mtb exposure, along with their lack of progression to TB disease. The TB-resister phenotype represents a robust level of years-long protection against Mtb infection, which may be occupational (e.g., TB nurses) or community based or happen in a TB household. As mentioned, besides the findings from the study by Erwin Schurr’s team in the *JCI* (16), several other correlates of protection have been identified, both innate (8) and adaptive (10, 12). One study has also reported genetic associations with the TB-resister phenotype (15).

TB resisters are probably a subset of early clearers who show protection over a shorter period of time (several months) in the context of a well-defined exposure (e.g., a TB household). In our cohort of 1,347 household contacts of patients with sputum-smear–positive TB in an urban setting in Indonesia, approximately 25% remained IGRA negative after 14 weeks and could be defined as early clearers, while 65% already tested IGRA positive at baseline and close to 10% converted from a negative to a positive IGRA by 14 weeks (5). IGRA conversion was associated with higher exposure (e.g., closer sleeping proximity to the index patient), while lack of IGRA conversion among household contacts was associated with presence of a bacillus Calmette-Guérin (BCG) scar. Interestingly, this protection by BCG (adjusted hazard ratio 0.56; 95% CI 0.40–0.77) was lower among household contacts with higher TB exposure. Furthermore, household contacts exposed to L2 strains (also known as Beijing strains) showed no evidence of BCG protection against IGRA conversion, suggesting vaccine escape (20). These associations became stronger with use of stricter IGRA cut-offs to improve the signal to noise ratio. As such, compared with the TB resister phenotype, early clearance reflects a more relative or dynamic protection against Mtb infection that can be overcome by high exposure or particular Mtb strains. Our lab has identified altered innate immune cell dynamics, higher cytokine production, and higher inflammatory proteins in IGRA mitogen tubes as innate immune correlates of early clearance (21), while another group has identified epigenetic marks linked to IGRA conversion (22). It is unclear if better vaccines or other interventions can further improve innate immunity to prevent an overwhelmed response to a high-level exposure. Similarly, studies need to evaluate whether there are wider strain-specific responses to BCG and recently developed TB vaccine candidates, influencing their population-level impact.

## Translating the findings to TB prevention

Better understanding of the multiple correlates of protection against Mtb infection through studies in TB resisters and early clearers can help advance efforts to boost protection against TB disease. Traditionally, TB-preventive studies have used symptomatic disease as a primary endpoint, but there are good reasons to include asymptomatic TB as well (23). We argue that Mtb infection should also be considered as an endpoint. The observational evidence that BCG vaccination protects against Mtb infection is strong (24), and integrated multiomics analysis has increased our understanding of related BCG-induced trained immunity (25), which, like β-glucan–induced trained immunity, protects against TB in experimental models (26, 27). Protection against Mtb infection and immunological, epigenetic, or metabolic correlates of this protection should therefore be included in TB vaccine studies.

Other interventions also offer promise. The landmark RATIONS trial in 2023 showed that nutritional supplementation of TB household contacts in India, with a combination of micro- and macronutrients, led to a 48% decrease (95% CI 21%–56%) in microbiologically confirmed TB (28), but the study did not evaluate whether nutritional support was effective against asymptomatic TB or the development of Mtb infection in the first place. A smaller trial conducted in New York in the 1940s showed an equally remarkable reduction in TB disease in household contacts with micronutrient (vitamin) supplementation alone (29). There is robust evidence linking nutrition with innate immunity, and therefore studies are needed to evaluate whether nutritional support will enhance protection against Mtb infection.

For now, evidence from multiple lines of enquiry is increasingly supportive of official confirmation of the early clearance phenotype and the stricter subset of TB resisters in the deepening understanding of the interaction between Mtb and *Homo sapiens*. Early clearance may occur in up to 25% of individuals from any one exposure event and could hold the secret to an optimized TB vaccine and a new generation of other preventive treatments in the fight against this most brutal of all pathogens.

## Version 1 04/01/2025

Electronic publication
